# Erratum: Vol. 68, No. 27

**DOI:** 10.15585/mmwr.mm6831a3

**Published:** 2019-08-09

In the report “*Vital Signs*: Surveillance for Acute Flaccid Myelitis — United States, 2018,” on page 609, in the first paragraph of the Results section, the fourth sentence should have read “Patients with illnesses classified as non-AFM were significantly older than were patients with confirmed AFM (median = **8.8** years [range = 1 month–78.1 years]; p<0.001) (Table 1).”

On page 611, in Table 1, for “Laboratory finding; Spine MRI performed,” the number of confirmed cases should have been “**233/233 (100)**,” and the P-value should have been “**0.10**.” In addition, for “Timing of preceding illness to onset of limb weakness, median days (range, IQR); Any respiratory illness,” the interquartile range (IQR) for noncases should have been “6.5 (0–28, **4–11.5**).”

